# 

**Published:** 1880-06

**Authors:** 


ORIGINAL COMMUNICATIONS: PAGE.

Testimony of Medical Experts, by Hon. Charles Beckwith, . . . . . . 469

CLINICAL REPORTS:

Injuries to the Cranial Bones—Trephining. Reported by S. G. Dorr, M. D., . . 475
Spontaneous Discharge of a Large Vesical Calculus from a Male. Reported by Charles G. Stockton, M. D., . . . 479

TRANSLATIONS:

Nervous Dyspepsia. From the Dutch by P. W. Van Peyma, M. D., . . . 481
The Bromide of Ethyl as a Local Anæsthetic. From the French by F. Peterson, M. D., - - . . . . . 483
Coto Bark in Diarrhea of Phthisis, . . 484

SELECTIONS:

The Audiphone and Kindred Instruments, by E. L. Holmes, M. D., . . . 484
Stretching of Sciatic, Digital, and Infraorbital Nerves, . . . . . . 487
Treatment of Leucocythœmia, by Alfred Carpenter, M. D., . . . . . . 489
Hepatic Anæmia, . . . . . . . 490
Rupture of the Duedenum from a blow on the Abdomen. . . . . . . . 492
Accidents observed to follow Thoracentesis, by Aspiration, &c., . . . . . . 494
Cough produced by Accumulations in the Ear, 495
Oxalate of Cerium as a Cough Remedy, . 496
Signs of Death by Drowning, . . . 496

PAGE.

Expectant Treatment in Caries of the Ankle in Children, . . . . . . 497
Vaccinium Classifolium aut Repens, . . 498
The Entire Uterus torn from a Puerperal Woman without Fatal Consequences, . 498
"Nothing in it," . . . . . . 499
Decrease in Bodily Weight after Epileptic Attacks, . . . . . . . . 499
The Treatment of Pott's Disease by the Plaster-of-Paris Jacket, . . . . . 500
Iron and Digitalis, . . . . . . 500
Liability of Hospitals in Malpractice Suits, 501
Rapid Cure for a Cold, . . . . . 501
Harvard Medical School, . . . . . 501
Per-Vaginal Enucleation of the Uterus, &c., by L. C. Lane, M. D., . . . . . 502

SPECIAL COMMUNICATIONS:

Is it Professional? . . . . . . 504

SOCIETY REPORTS:

Thirteenth Annual Meeting of the Medical Association of Central New York, . 506

EDITORIAL:

Two Points in the History of a Case, . . 509
The American Medical Association—Thirty-First Annual Meeting, . . . . . 511
United States Postal Laws and our Subscribers, . . . . . . . . 512

REVIEWS, . . . . . . . 513
Books and Pamphlets received, . . . 516

